# Bacterial Coinfections Increase Mortality of Severely Ill COVID-19 Patients in Saudi Arabia

**DOI:** 10.3390/ijerph19042424

**Published:** 2022-02-19

**Authors:** Abdulaziz Alqahtani, Edrous Alamer, Mushtaq Mir, Ali Alasmari, Mohammed Merae Alshahrani, Mohammed Asiri, Irfan Ahmad, Abdulaziz Alhazmi, Abdullah Algaissi

**Affiliations:** 1 Clinical Laboratory Sciences, College of Applied Medical Sciences, King Khalid University, Abha 61321, Saudi Arabia; ayali@kku.edu.sa (A.A.); mmir@kku.edu.sa (M.M.); masiri@kku.edu.sa (M.A.); imahmood@kku.edu.sa (I.A.); 2 Emerging and Epidemic Infectious Diseases Research Unit, Medical Research Center, Jazan University, Jazan 45142, Saudi Arabia; ealamer@jazanu.edu.sa (E.A.); abalhazmi@jazanu.edu.sa (A.A.); 3 Department of Medical Laboratory Sciences, Faculty of Applied Medical Sciences, Jazan University, Jazan 45142, Saudi Arabia; 4 Asir Central Hospital, Abha 61321, Saudi Arabia; ali.ksa2008@gmail.com; 5 Department of Clinical Laboratory Sciences, Faculty of Applied Medical Sciences, Najran University, 1988, Najran 61441, Saudi Arabia; mmalshahrani@nu.edu.sa; 6 Microbiology and Parasitology Department, College of Medicine, Jazan University, Jazan 45142, Saudi Arabia

**Keywords:** COVID-19, coinfection, bacteria, SARS-CoV-2, Saudi Arabia

## Abstract

Coronavirus disease 19 (COVID-19) is an ongoing global pandemic that is caused by severe acute respiratory syndrome coronavirus 2 (SARS-CoV-2). The severity and mortality rates of COVID-19 are affected by several factors, such as respiratory diseases, diabetes, and hypertension. Bacterial coinfections are another factor that could contribute to the severity of COVID-19. Limited studies have investigated morbidity and mortality due to microbial coinfections in COVID-19 patients. Here, we retrospectively studied the effects of bacterial coinfections on intensive care unit (ICU)-admitted patients with COVID-19 in Asir province, Saudi Arabia. We analyzed electronic medical records of hospitalized patients with COVID-19 at Asir Central Hospital. A total of 34 patients were included, and the clinical data of 16 patients infected with SARS-CoV-2 only and 18 patients coinfected with SARS-CoV-2 and bacterial infections were analyzed in our study. Our data showed that the length of stay at the hospital for patients infected with both SARS-CoV-2 and bacterial infection was 35.2 days, compared to 16.2 days for patients infected with only SARS-CoV-2 (*p* = 0.0001). In addition, higher mortality rates were associated with patients in the coinfection group compared to the SARS-CoV-2-only infected group (50% vs. 18.7%, respectively). The study also showed that gram-negative bacteria are the most commonly isolated bacteria in COVID-19 patients. To conclude, this study found that individuals with COVID-19 who presented with bacterial infections are at higher risk for a longer stay at the hospital and potentially death. Further studies with a larger population are warranted to better understand the clinical outcomes of COVID-19 with bacterial infections.

## 1. Introduction

Coronavirus Disease 2019 (COVID-19) is an ongoing global pandemic disease that is caused by severe acute respiratory syndrome coronavirus 2 (SARS-CoV-2) [1]. Since late 2019, SARS-CoV-2 has resulted in more than 274 million confirmed cases, and approximately 5.4 million deaths have been reported globally [2]. SARS-CoV-2 infections can result in a wide range of clinical manifestations, which can be asymptomatic, mild, or symptomatic, resulting in severe pneumonia and multiorgan involvement that may lead to death [3]. In several cases, COVID-19 may result in mild or moderate manifestations; however, patients with comorbidities may require intensive care and mechanical ventilation, which may predispose them to secondary, opportunistic, and hospital-acquired infections (HAIs) [4,5].

Variations in the symptoms among COVID-19 patients can be mediated by several factors, including, but not limited to, health conditions, age, sex, and other biological factors [6]. Viral infection of the lungs can dampen the immune system, which may result in alterations in the population and functions of respiratory microbiota, which may predispose hosts to secondary bacterial infection [7,8,9,10,11]. Overall, bacterial coinfections have been found to complicate viral respiratory infections and are associated with serious infection outcomes [12]. Secondary bacterial coinfection was identified as one of the major causes of mortality during past influenza pandemics [8,9,10,11]. In the H1N1 influenza pandemic of 2009, almost 30% of the cases were found to have bacterial coinfection, and more importantly, this occurred despite the initiation of antibiotic treatment [7,13]. It has been reported that secondary coinfections were the major mediators of the high morbidity and mortality in influenza-infected patients [10,14]. Recently, bacterial coinfection in COVID-19 patients was also reported in several studies among different countries [5,15,16,17,18]. The prevalence of bacterial coinfections among COVID-19 patients ranged from 12.4 to 50% [5,16,19,20,21,22,23]. For instance, a multicentral study from China reported that bacterial coinfection was detected in almost 34.5% of critically ill patients [24]. Concerningly, the rate of coinfections in those critical patients occurred even though most of the patients (92.9%) had received antibiotic treatments [24]. Although it is not fully clear yet whether the outcomes of COVID-19 patients are mainly worsened by secondary bacterial infections, data from past seasonal flu and other past influenza pandemics suggested that bacterial coinfections can worsen viral diseases [25,26,27,28]. Of note, almost 30% of patients infected with SARS-CoV during the 2003 outbreak presented secondary bacterial infections that were positively linked with the severity of the disease [29,30]. In general, almost 2–65% of past seasonal influenza cases presented with bacterial coinfections that were associated with high morbidity and mortality [25,26,27]. Severe cases of COVID-19 showed a reduction in the lymphocyte count, with a marked reduction in CD4 and CD8 T cells [31,32]. Such immune dysregulation may predispose patients to coinfection, which was diagnosed in almost 50% of patients who died from COVID-19 [33]. Several studies reported that most secondary coinfections among COVID-19 patients were HAIs rather than community-acquired infections, as most of the secondary infections were detected 48 h or more after hospital admission [16,34]. Therefore, the aim of our study was to determine the prevalence and outcomes of nosocomial bacterial infections in patients with COVID-19 in Saudi Arabia and the most common bacterial pathogens that are associated with SARS-CoV-2 infection.

## 2. Materials and Methods

### 2.1. Study Design and Participants

This is a retrospective study that included patients who were admitted to the ICU unit between 1 April 2020 and 1 June 2020, Asir Central Hospital (574 beds), Abha, Saudi Arabia. All patients were diagnosed with COVID-19 using RT-PCR (reverse transcription polymerase chain reaction) of nasopharyngeal and throat swabs to detect SARS-CoV-2 were included. We excluded patients who were admitted to other wards than the ICU, those who were admitted to the ICU outside of our study period, and ICU patients who were not diagnosed with COVID-19 using RT-PCR.

### 2.2. Ethical Approval

Ethical approval for conducting this study was obtained from the Research Ethics Committee at King Khalid University, Saudi Arabia (reference number; ECM#2021-5821, date 25 October 2022). Patients’ informed consents were waived by the Ethics Committee on a special request due to the retrospective nature of the study.

### 2.3. Data Collection

Patients’ data were collected from electronic medical records accessed from the laboratory department of Asir Central Hospital, Abha, Saudi Arabia. The data collected included patients’ age, gender, length of hospital stay, hematological and biochemical results, and microbiological data, including bacterial samples (blood, sputum, lung lavage, endotracheal tube, urine, throat swab, and bronchial secretion). Organisms’ identification and antibacterial susceptibility testing were performed using MiscroScan WalkAway (Beckman Coulter Inc; Carlsbad, CA, USA). Antibacterial susceptibility testing was performed using gram-positive (Pos Breakpoint Combo 28) and gram-negative (Neg Breakpoint Combo 50) MIC panels, and the results were interpreted in compliance with CLSI methods as recommended by the infrastructure’s instructions [35]. The data collection included only samples that were collected from patients ≥ 48 h after admission to exclude potential community-acquired infection. All hematological and biochemical data were collected from tests performed at the time of suspicion of secondary infections.

### 2.4. Data Analysis

Statistical analysis was performed using GraphPad Prism (Version 9.2.0) (GraphPad Software; San Diego, CA, USA). Descriptive statistics were also reported for the collected data. A *t*-test was performed to analyze two independent data groups. A *p*-value < 0.05 was considered statistically significant.

## 3. Results

### 3.1. Patients’ Characteristics

Patients’ demographic and clinical characteristics are shown in Table 1. The total number of COVID-19 patients who required ICU admission at Asir Central Hospital, Abha, between 1 April 2020 and 1 June 2020 was 34 patients.

The average age of patients was 64 years, and 79.5% of patients were male. The length of patients’ stay in the ICU ranged from 1 to 55 days, with a mean of 25.7 days. A total of 35.2% of COVID-19 patients who were admitted to the ICU eventually died. All ICU-admitted COVID-19 patients received bacterial detection testing when secondary infection was suspected. Samples were collected from locations according to the clinical manifestations of the bacterial infection, including blood, sputum, lung lavage, endotracheal tube, urine, throat, and bronchial secretions (Table 1). Almost 53% of the patients with COVID-19 who were admitted to the ICU were diagnosed with bacterial coinfection when tested ≥ 48 h following ICU admission (Table 1). Almost 50% of patients with bacterial coinfection succumbed to death, compared to only 18.7% of patients with SARS-CoV-2 infection only (Table 1). Furthermore, a significant increase in the length of ICU stay was reported among patients with bacterial infection compared to COVID-19-infected only, with stays of 35.2 and 16.2 days, respectively. Next, we analyzed biochemical and hematological markers that were immediately requested when a secondary bacterial coinfection was suspected (Table 2).

A *t*-test analysis showed a significant decrease in several parameters of coinfected patients, including random blood sugar (RBS), red blood cells (RBCs), hemoglobin, hematocrit, mean corpuscular volume (MCV), and platelets, when compared to patients with COVID-19 only. Urea was significantly increased in bacterial-infected patients compared to non-bacterial-infected patients. Furthermore, other parameters revealed different results in the coinfected group compared to patients with COVID-19 infection only; however, the differences in these parameters were not significant (Table 2).

### 3.2. Bacterial Isolation and Antimicrobial Resistance Patterns

Different bacterial species were identified among COVID-19 patients, including four Gram-positive organisms (*Staphylococcus epidermidis*, *Enterococcus faecium*, *methicillin-resistant Staphylococcus aureus* (*MRSA*), *Enterococcus faecalis*) and five gram-negative bacteria (*Acinetobacter baumannii*, *Klebsiella pneumoniae*, *Proteus mirabilis*, *E. coli*, and *Enterobacter* species). These bacterial isolates were detected with different frequencies among the ICU patients (Table 3). Overall, bacterial pathogens were isolated more frequently from blood (12), followed by sputum and endotracheal tube (5), and urine (3) (Table 3).

Of note, 44% of the coinfected patients (8 out of 18 patients) showed positive bacterial culture in more than one location, and 50% of these patients tested positive for multiple bacterial species (patients ID: 1, 2, 3, and 7) (Table 4).

**Figure 1:**
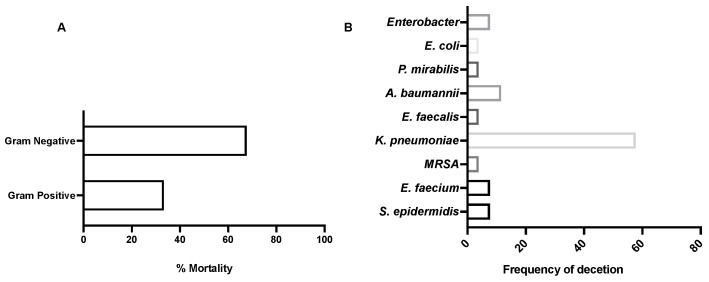
Bacteria isolated from COVID-19 patients: (**A**) percentage of mortality among COVID-19 patients infected with either gram-positive or gram-negative bacteria; (**B**) frequency of the type of detected bacterial isolates from COVID-19 patients.

Overall, we found that almost 70% of the coinfected patients who eventually died were infected with gram-negative organisms (Figure 1A). Further, we found that *K. pneumoniae* was the most frequently reported organism among all other bacterial isolates, followed by *A. baumanni* isolates, at 57.69% and 11.5%, respectively (Figure 1B).

We extended our analysis further to study the antimicrobial resistance patterns of the gram-negative isolated bacteria. Patients’ records showed that wide antibiotic panels (11–21 antibiotics) were used to perform the antimicrobial sensitivity screening for the isolated bacteria (Table 5). The rates of resistance for gram-negative bacteria for each tested antibiotic are shown in Table 6. Of note, six isolates (50%) of *K. pneumoniae* were phenotypically reported as carbapenem-resistant Enterobacteriaceae (CRE), and one isolate (50%) of *Enterobacter* spp. was phenotypically reported as extended-spectrum beta-lactamase (ESBL).

## 4. Discussion

Viral infections of the respiratory tract have long been linked to the risk of secondary bacterial infections. Bacterial coinfections were considered a major cause of death in previous influenza pandemics [5,7,8]. Viral lung infection dysregulates the host’s immunity, altering the composition and functions of the respiratory microbiome, which may predispose the host to secondary infection [13]. More recently, a few studies have reported the incidence of secondary bacterial infection in critically ill COVID-19 patients, which has been linked to a noticeable surge in COVID-19 severity and mortality [5,16,18,19,20,23,36]. Here, this study aimed to evaluate the burden of bacterial coinfections among ICU-admitted COVID-19 patients in Saudi Arabia. The study analyzed the medical records of all patients admitted to the ICU at Asir Central Hospital, Abha, Saudi Arabia, between 1 April 2020 and 1 June 2020.

In the current study, we showed that 53% of the study subjects had secondary bacterial infections when evaluated ≥ 48 h after ICU admission, indicating a high prevalence of nosocomial bacterial infections among COVID-19 patients in Saudi Arabia. Several studies have reported the incidence of bacterial coinfection among hospitalized COVID-19 patients in different countries [5,16,18,19,20,23,36]. Furthermore, most of the reported secondary infections were thought to be HAIs rather than community-acquired infections [16,34]. For instance, a recent study showed that the incidence of community-acquired infection was very low (5.5%); however, the proportion of pathogens detected increased with the length of ICU stay, consisting mainly of gram-negative bacterial infections [16]. HAIs can occur as a result of ventilator-associated pneumonia, central-line-associated bloodstream infections, and catheter-associated urinary tract infections [36]. The prevalence of bacterial coinfections among COVID-19 patients has been reported in several previous studies, ranging from 12.4% to 50% [5,16,19,20,21,22,23]. Our study also showed that the length of ICU stay was significantly higher in patients with secondary bacterial infection compared to patients with no detected bacterial infection (*p* = 0.0001). Our data agree with several reports that showed that secondary infection extended the length of ICU stay, which was associated with a higher risk of death [5,18]. Furthermore, consistent with several previous reports [5,18], coinfection among our study subjects increased the risk of death. A recent study investigated the risk of death due to secondary bacterial infections in patients with COVID-19 [5]. The study found that coinfected patients reported a higher mortality rate compared to patients with COVID-19 only, at 61% and 13%, respectively [5]. Another previous study on COVID-19/bacterial coinfection concluded that patients with coinfections were more likely to die in the ICU compared to non-bacterial-colonized patients [16]. Based on our data and the previous results, it seems that secondary bacterial infections are the major cause of the high mortality rate among our COVID-19 patients. However, it is difficult to determine the extent of severity, as well as the direct pathological impact of the secondary coinfections on patients’ mortality in our study subjects, as other factors may also contribute to mortality, e.g., comorbidities that have not been reported for the examined COVID-19 patients. Bacterial infections during a COVID-19 infection can disturb several hematological and biochemical parameters, impacting the general clinical condition [37]. The present study found significant changes in the hematological profile, including RBCs, HB, hematocrit, MCV, and urea with RBS, in patients with positive cultures compared to those with negative bacterial cultures. Several reports have shown dysregulation in different laboratory findings [5,18]. A previous report showed that patients with coinfection showed dramatic changes in levels of leucocyte count, creatinine, hemoglobin, and urea in patients with positive bacterial cultures compared to those with negative bacterial cultures [5]. However, the clinical relevance of these changes needs to be investigated. Among all detected bacterial isolates, we showed that more gram-negative bacteria were identified among our study subjects (55.6%) (Table 3 and Table 6). Our data are also consistent with a previous report that showed that gram-negative bacteria are the most common organisms isolated from COVID-19, at 85.5% of the total coinfected patients [38]. According to antibiotic susceptibility testing, our study showed that some of the gram-negative bacteria were reported as ESBL and CRE, which are highly resistant to antibiotics, including last-resort antibiotics such as Meropenem and Colistin (Table 6). Recently, an overall considerable rise in the incidence of ICU infections caused by MDR gram-negative bacteria has been reported. This rise has resulted in prolonged hospitalizations, which were also associated with high morbidity and mortality [38]. We also showed that, among all the gram-negative isolates, *K. pneumoniae* was the most frequently reported infection (57.69%), and almost 90.4% of the tested antibiotics showed ineffectiveness against this isolate (Table 6). In general, *K*. *pneumoniae* with MDR is becoming a global threat, as it has high virulence factors and shows a low response to treatment [39]. Our data also agree with a previous report that showed that *K. pneumoniae* was one of the most frequently isolated bacteria in COVID-19 patients [5,23,36,39]. Jie Li et al. showed that *K. pneumoniae* was one of the top three isolated organisms that also showed high resistance rates to several antibiotics [40].

Although our study provided important findings on the impact of secondary bacterial infections on patients with COVID-19, it has a few limitations. First, the sample size of our study is small. We analyzed the medical record of 34 patients; thus, it is difficult to determine whether our findings can be applied to all COVID-19 patients. Second, the data were collected from a single hospital; thus, the identified bacteria might reflect a site-specific microbiological profile. Third, this study was not able to analyze other clinical information, such as patients’ comorbidities, which may have a great impact on disease severity and mortality. Fourth, the study provided some hematological and biochemical findings, comparing non-bacterial-infected and bacterial-coinfected patients at a single point in time (tests performed when coinfection is suspected), and no follow-up findings were presented. Finally, we could not confirm the genotypic resistance profiles of bacterial isolates in our study, despite their phenotypical appearance as highly resistant bacteria (such as ESBL or CRE). Therefore, multicentral future studies with a larger sample size are warranted to investigate the relationship between secondary bacterial infections and COVID-19.

## 5. Conclusions

In conclusion, to our knowledge, this is the first study to assess the burden of secondary bacterial coinfection among COVID-19 patients in Saudi Arabia. We showed a high prevalence of hospital-acquired bacterial infections among our ICU-admitted COVID-19 patients (53%) between 1 April 2020 and 1 June 2020. Bacterial colonization in our study increased the length of ICU hospitalization and the mortality rate. Gram-negative bacteria were shown to be the most commonly prevalent organisms among the examined ICU patients, which also showed significant resistance to antibiotics. Therefore, more studies are needed to investigate the impact of antimicrobial-resistant bacteria on the clinical outcomes of COVID-19 patients. Although the sample size of our study is small, data from this study, like others [5,16,18,19,20,23,36], may be useful in understanding the burden of bacterial hospital-acquired infections among COVID-19 patients, and future studies should include a larger population from different centers to investigate the impact of HAIs on COVID-19 patients. Further, our study has important implications on infection control and prevention in hospitals, which need to be improved to limit the chance of bacterial coinfection. Another important implication of the study is on diagnostic testing protocols for COVID-19 patients. As our data showed that bacterial coinfection is not infrequent in hospitalized COVID-19 patients, it could be beneficial to run panel testing on those patients and determine whether there is a coinfection. Lastly, clinical management of COVID-19 patients should also consider the assessment of coinfections so that treatment for both COVID-19 and bacterial infection can be administered.

## Figures and Tables

**Table 1:** Characteristics and microbiologic investigations of the study patients.

Characteristics	All Patients (*n* = 34)	SARS-CoV-2 (*n* = 16) (47%)	SARS-CoV-2/Bacterial Coinfection (*n* = 18) (53%)	*p*-Value
Age (years), median	64	66	63	0.6892
Gender, n (%)				
Male	27 (79.5)	13 (81.25)	14 (77.7)	0.8315
Female	7 (20.5)	3 (18.75)	4 (22.3)	
Mortality, (%)	35.2	18.7	50	0.0589
Length of stay in ICU, days, (mean)	25.7	16.2	35.2	0.0001 *
Sample site	Bacterial investigations undertaken (*n*)			
Blood	14	1	11	
Sputum	8	3	5	
Lung lavage	1	0	1	
Endotracheal Tube	7	2	5	
Urine	10	7	3	
Throat Swab	4	3	1	
Bronchial Secretion	1	1	0	

* Statistically important.

**Table 2:** Some laboratory parameters for the study subjects.

Parameter	Patients with No Bacterial Infection (*n* = 16) (47%)	Patients with Bacterial Infection (*n* = 18) (53%)	*p*-Values
ALT (U/L)	72.3 ± 69.9	94.04 ± 126.3	0.5867
AST (U/L)	104.7 ±174.1	132 ± 274	0.7563
Urea (mg/dL)	70.96 ± 73.2	132.3 ± 108.6	0.0422 *
Random blood sugar (RBS) (mg/dL)	249.8 ±174.2	153.5 ± 74.7	0.0428 *
Creatinine (mg/dL)	1.882 ± 2.6	1.858 ± 1.38	0.9734
Potassium Mmol/L	4.581 ±1.23	4.282 ± 0.69	0.3971
Activated Partial Thromboplastin Time (APTT) (Seconds)	50.79 ±46.22	65.34 ± 39.36	0.3852
Prothrombin Time (Seconds)	19.44 ± 14.75	18.78 ± 9.86	0.8905
WBCs (10^3^/μL)	13.26 ± 11.9	15.64 ± 18.76	0.6675
RBCs (10^6^/μL)	4.308 ± 1.1	2.920 ± 0.68	<0.0001 *
Hemoglobin (g/dL)	12.63 ± 5.58	8.100 ± 1.88	0.0028 *
Hematocrit (%)	39.01 ± 14.59	26.04 ± 6.39	0.0017 *
MCV (fl)	79.85 ± 15.18	89.08 ± 7.19	0.0276 *
MCH (pg)	26.78 ± 2.8	27.74 ± 1.9	0.2466
MCHC (g/dL)	44.55 ± 5.3	31.26 ± 2.1	0.2799
Platelets (10^3^/μL)	209.0 ± 125.1	106.2 ± 86	0.0082 *
RDW (fL)	45.14 ± 12.6	54.08 ± 13.3	0.0543
Neutrophils (%)	72.32 ± 26.6	82.72 ± 15.3	0.1664
Absolute Neutrophils (10^3^/UL)	8.946 ± 7.6	14.08 ± 18.6	0.3118
Lymphocytes (%)	14.62 ± 17	8.144 ± 8	0.1590
Absolute Lymphocyte Count (10^3^/μL)	2.348 ± 4.2	0.7372 ± 0.59	0.1243
Monocytes (%)	5.162 ± 3.7	4.017 ± 2.6	0.3054
Absolute monocytes (10^3^/μL)	0.6500 ± 0.68	0.4933 ± 0.40	0.4141
Eosinophils (%)	1.609 ± 2.7	4.817 ± 13.7	0.3661
Basophils (%)	0.5775 ± 0.93	0.3118 ± 0.3	0.2742
Neutrophil Lymphocyte Ratio (NLR)	9.4 ± 68.1	16.6 ± 59.67	0.7431

* Statistically important.

**Table 3:** Frequency of bacterial species detected in different anatomical locations of the 18 critically ill COVID-19 patients.

Bacterial Species (*n* = 9)	Bacteria	Sample Site (*n* = Frequency)
Gram-positive (n = 4, 44.4%)	*Staphylococcus epidermidis*	Blood (2)
	*Enterococcus faecium*	Blood (3)
	*MRSA*	Blood (1)
	*Enterococcus faecalis*	Blood (1)
Gram-negative (n = 5, 55.6%)	*Acinetobacter baumannii*	Blood (2), throat swab (1)
	*Klebsiella pneumoniae*	Blood (3), sputum (4), endotracheal tube (3), urine (2)
	*Proteus mirabilis*	Lung lavage (1)
	*E. coli*	Urine (1)
	*Enterobacter*	Sputum (1), endotracheal tube (1)

**Table 4:** Patients with bacteria detected in different locations.

Patients ID (n = 8 (44.4%))	Sample Site	Bacteria
1	Blood	*Acinetobacter baumannii*
	Sputum	*Klebsiella pneumoniae*
2	Blood	*Staphylococcus epidermidis*
	Lung lavage	*Proteus mirabilis*
3	Blood	*Enterococcus faecalis*
	Endo Tracheal Tube	*Acinetobacter baumannii*
4	Blood	*Klebsiella pneumoniae*
	Sputum	*Klebsiella pneumoniae*
5	Blood	*Klebsiella pneumoniae*
	Sputum	*Klebsiella pneumoniae*
6	Blood	*Klebsiella pneumoniae*
	Endo Tracheal Tube	*Klebsiella pneumoniae*
7	Urine	*E. coli*
	Endo Tracheal Tube	*Klebsiella pneumoniae*
8	Urine	*Klebsiella pneumoniae*
	Endo Tracheal Tube	*Klebsiella pneumoniae*

**Table 5:** Antibiotics panels used for antibiotic susceptibility screening of gram-positive and gram-negative bacteria in the study patients.

Antibiotics Used for Screening Gram-Positive Bacteria	Antibiotics Used for Screening Gram-Negative Bacteria
Ampicillin	Amikacin
Azithromycin	amoxicillin and clavulanic acid
Ciprofloxacin	Ampicillin/Sulbactam
Clindamycin	Aztreonam
Daptomycin	Cefepime
Erythromycin	Cefotaxime
Fosfomycin	Cefoxitin
Fusidic Acid	Ceftazidime
Gentamicin	Ceftriaxone
Levofloxacin	Cefuroxime
Linezolid	Ciprofloxacin
Moxifloxacin	Colistin
Mupirocin	Ertapenem
Nitrofurantoin	Gentamicin
Oxacillin	Imipenem
Penicillin	Levofloxacin
Rifampin	Meropenem
Sulfamethoxazole and trimethoprim	Minocycline
Synercid	Moxifloxacin
Teicoplanin	Nitrofurantoin
Tetracyclin	Piperacillin/tazobactam
Tigecycline	Sulfamethoxazole and trimethoprim
Vancomycin	Tigecycline
	Tobramycin

**Table 6:** Rate of resistance for gram-negative bacteria.

Antibiotics/Isolates (n)	*A. baumannii* (4)	*Enterobacter* spp. (2)	*K. pneumoniae* (12)	*E. coli* (1)	*P. mirabilis* (1)
Gentamicin	100%	50%	75%	100%	100%
Amikacin	75%	50%	75%	0%	0%
Tobramycin	75%	100%	100%	0%	0%
amoxicillin and clavulanic acid	100%	100%	100%	100%	0%
Ampicillin/Sulbactam	25%	100%	100%	100%	0%
Cefepime	100%	100%	100%	100%	0%
Cefotaxime	100%	100%	100%	100%	0%
Cefoxitin	100%	100%	100%	100%	0%
Ceftazidime	100%	100%	100%	100%	0%
Ceftriaxone	100%	100%	100%	100%	0%
Cefuroxime	100%	100%	100%	100%	0%
Piperacillin/tazobactam	100%	100%	92%	0%	0%
Ciprofloxacin	100%	50%	100%	100%	0%
Levofloxacin	100%	50%	100%	100%	100%
Ertapenem	100%	50%	100%	0%	0%
Imipenem	100%	50%	50%	0%	0%
Meropenem	100%	50%	100%	0%	0%
Tigecycline	50%	0%	0%	0%	0%
Colistin	0%	50%	50%	0%	0%

## Data Availability

The data presented in this study are available on request from the corresponding author.
